# Metachronous multiple gastrointestinal stromal tumors and adenocarcinoma of the colon: A case report

**DOI:** 10.3892/ol.2014.2266

**Published:** 2014-06-18

**Authors:** YONG-PENG WANG, YI LI, CHUN SONG

**Affiliations:** 1 Department of Colorectal Surgery, The Liaoning Provincial Tumor Hospital, Shenyang, Liaoning 110042, P.R. China; 2 Department of Pathology, The Liaoning Provincial Tumor Hospital, Shenyang, Liaoning 110042, P.R. China

**Keywords:** colonic adenocarcinoma, gastrointestinal stromal tumor, metachronous neoplasms

## Abstract

Synchronous or metachronous occurrence of gastrointestinal stromal tumors (GISTs) and other primary gastrointestinal neoplasms has previously been reported. However, to the best of our knowledge, there are few studies regarding metachronous multiple GISTs and adenocarcinoma of the colon. The current case of an 80-year-old male patient who underwent a laparoscopic right hemicolectomy for colonic adenocarcinoma, located in the ascending colon, is presented. Twenty-one months after receiving the laparoscopic right hemicolectomy, two new disc-like bulge lesions in the descending colon and rectosigmoid were identified during an endoscopic follow-up examination, and a segmental bowel resection was performed. The final diagnosis of multiple colonic GISTs was established as a result of histopathological examination and immunohistochemistry.

## Introduction

Gastrointestinal stromal tumors (GISTs) are a common mesenchymal tumor of the gastrointestinal tract. GISTs were mistaken for leiomyosarcoma and other mesenchymal sarcomas prior to 1983 and have an incidence of ~1–2/100,000 worldwide (1–4). The majority of GISTs are located in the stomach and small intestine, while ~5% of the cases reported are located in the colon and rectum (5).

Previously, the synchronous occurrence of GISTs and other primary gastrointestinal malignancies has been reported in the literature (6–7). However, to the best of our knowledge, there are few studies regarding either synchronous or metachronous GISTs (particularly multiple GISTs), as well as adenocarcinoma of the colon and rectum. In the current report, we present the case of a patient who exhibited metachronous multiple colonic GISTs as well as adenocarcinoma of the colon.

## Case report

In June 2008, an 80-year-old male patient was admitted to the Department of Colorectal Surgery at The Liaoning Provincial Tumor Hospital (Shenyang, China) complaining of anemia and increasing paroxysmal abdominal pain during the previous month prior to admission. The patient’s medical history was unremarkable. On physical examination, the patient’s abdomen was flat and soft without tenderness and no mass was palpated. A blood count test revealed anemia, while the levels of carcinoembryonic antigen (CEA) and carbohydrate antigen (CA)19-9 were 14.14 ng/ml and 28.9 U/l, respectively. The chest X-ray was unremarkable and an abdominal computed tomography (CT)-scan, conducted to establish the tumor staging, demonstrated that there were no sites of distant metastasis. A fiberoptic colonoscopy showed an irregular bulging mass in the ascending colon, which occupied half of the intestinal lumen, however, no other abnormalities were detected in the remaining regions of the colon and rectum. Histological examination of the biopsy specimen revealed a well-differentiated adenocarcinoma.

**Figure 1:**
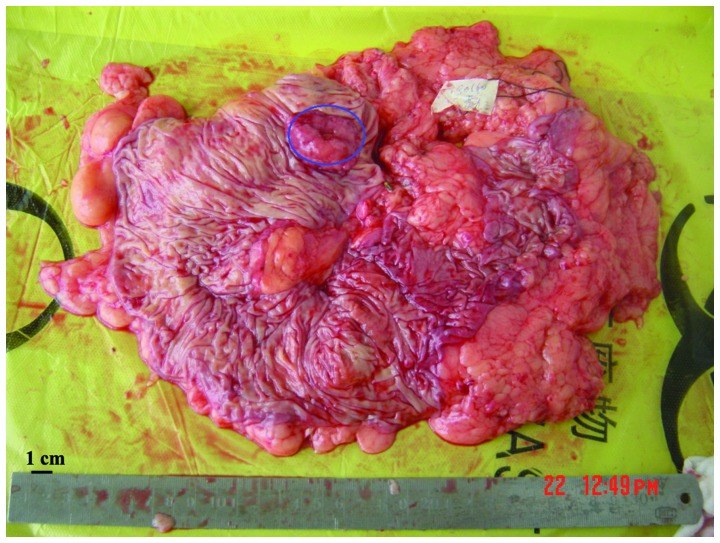
Gross sample of the tumor mass of the ascending colon; the lesion is encircled. Colonic multiple stromal tumors were not anticipated during the resection of the ascending colon, however, the tumor mass is clearly demonstrated.

**Figure 2:**
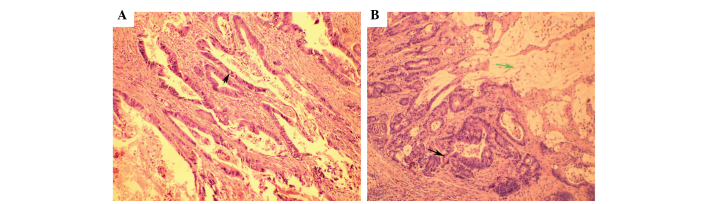
Hematoxylin and eosin staining of the colonic adenocarcinoma. Cancer cells showed a glandular arrangement, with large nuclear hyperchromatism and pleomorphism, and mitotic division was visible (green arrow). Certain cancer cells were observed to excrete mucus (black arrow). Magnification, ×200.

A laparoscopy assisted right hemicolectomy was subsequently performed. During surgery, a tumor mass, measuring 3×2 cm, which was invading the serous membrane was detected in the ascending colon; there was no evidence of lymph node metastasis (Fig. 1). Histological examination of the whole resected specimen demonstrated the combined presence of a moderately differentiated adenocarcinoma and a mucinous adenocarcinoma, which were located at the ascending colon and invading the subserous layer of the colon (Fig. 2). None of the 16 resected lymph nodes contained metastasis. According to the seventh edition of the tumor, node, metastasis (TNM) staging classification (2009) of colorectal cancer from the American Joint Committee on Cancer, the pathological stage of the tumor was determined to be T3N0M0. Following surgery, the patient received oral capecitabine (Xeloda^®^; F. Hoffmann-La Roche, Basel, Switzerland) as adjuvant chemotherapy at a dose of 1,250 mg/m^2^ twice daily, administered on days 1–14 every 21 days over 24 weeks.

**Figure 3:**
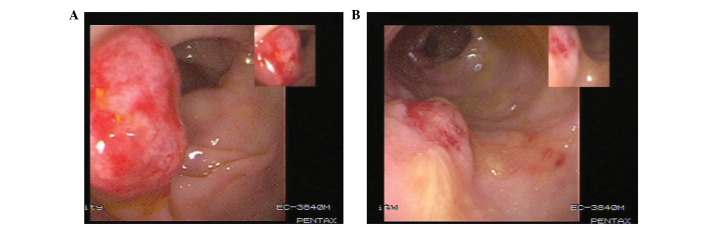
Colonoscopy observation. (A) A lesion in the descending colon. (B) An additional lesion in the rectosigmoid. A preoperative fiber colonoscopy to identify the stromal tumor showed comparable morphology to the colonic adenocarcinoma.

**Figure 4:**
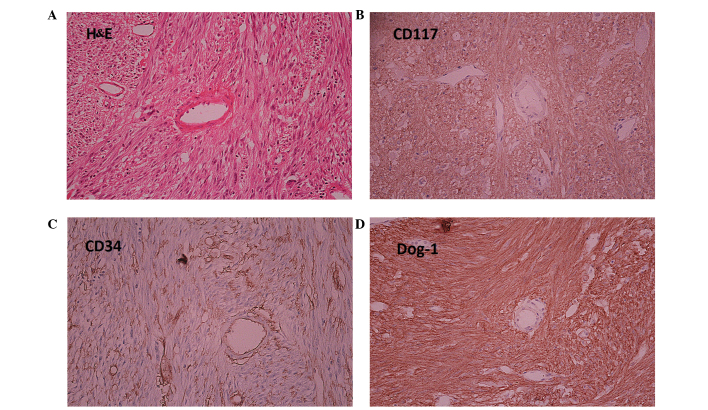
H&E and immunohistochemical staining of gastrointestinal stromal tumors (magnification, ×400). (A) H&E staining demonstrated a different cellular morphology compared with the adenocarcinoma as it was spindle-shaped. Immunohistochemical analysis showed positive staining for (B) CD117, (C) CD34 and (D) Dog-1. H&E, Hematoxylin and eosin; CD, cluster of differentiation; Dog-1, discovered on GIST-1.

A regular follow-up regime included a complete physical examination, basic serum chemistry, a chest X-ray, an abdominal ultrasound or CT-scan, and assessments of CEA and CA19-9 levels; this regime was performed every three months for two years. A fiberoptic colonoscopy was performed annually following treatment. In April 2010, two disc-like bulging lesions were identified in the descending colon and rectosigmoid during the follow-up colonoscopy. This included one lesion, which was a distance of 40 cm from the anus at the descending colon and measured 1.5×1.5 cm. The other lesion was located at the rectosigmoid and measured 1.2×1.2 cm (Fig. 3). An exploratory laparotomy followed by a segmental bowel resection was performed. The two lesions infiltrated the basement membrane and the submucosa. Pathological examination revealed that the two colonic lesions were mesenchymal cell tumors with mitotic activity, which were less than five mitoses per 50 high-power fields. Immunohistochemistry of the lesions were positive for cluster of differentiation (CD)117, CD34 and discovered on GIST-1 (Fig. 4). Routine treatment was administered following the surgery and the patient recovered well. Written informed consent was obtained from the patient and the patient’s relatives for publication of this case report.

## Discussion

Previous studies have demonstrated that ~20% of patients with GISTs develop other types of cancer, with the predominant GIST-associated cancers being gastrointestinal (47%), lymphoma/leukemia (7%), prostate (9%) and breast (7%) (6–7). However, to the best of our knowledge, a coexistence of metachronous multiple GISTs and colorectal carcinoma has not previously been described. In the present case report, a patient with multiple GISTs, which were identified during a routine follow-up colonoscopy examination 21 months after a colectomy, is presented.

Despite recent progress regarding the diagnosis and treatment of GISTs, little is currently known concerning the rare case of synchronous or metachronous GISTs along with tumors of different histogenesis. The majority of previous case reports describe small GISTs that were discovered during surgical procedures for another primary malignancy (8). In the present case, two lesions could have been incorrectly diagnosed as a reappearance of colon cancer, as the second colonoscopy and biopsy prior to surgery were indicative of a case of poorly differentiated adenocarcinoma; furthermore, the patient had a history of cancer of the ascending colon. Therefore, it is important to be aware that lesions, which are discovered during a postoperative colonoscopy following colorectal cancer surgery, may be another primary tumor of different histopathology rather than a recurrence.

As a result of postoperative pathological examination and immunohistochemical analysis of the relevant factors, including CD117, the diagnosis was finally determined to be colonic multiple GISTs. Based on the location, size and number of mitotic figures, and according to the improved National Institute of Health risk classification (9), the patient was considered to have a low-grade tumor. Generally, GISTs are sporadic, however, they may be detected as multiple lesions in familial forms and associated with type 1 neurofibromatosis (10). Furthermore, the majority of small GISTs are asymptomatic and, when they present alone, mimic other neoplastic conditions, which complicates the diagnosis (11,12). In the present case, the preoperative pathology was not consistent with the postoperative pathology as the colonoscopic biopsy obtained samples which were too small and the morphous was different as it exhibited an increased number of intestinal epithelial cells, which resulted in a diagnosis of poorly differentiated or undifferentiated adenocarcinoma rather than GISTs.

As the natural history of GISTs is unknown, the method for the effective management of GISTs that are <2 cm in size remains elusive. However, the present case indicates that a resection may be essential for a colonic cancer patient, when the endoscopic diagnosis of new lesions in the colon, during an endoscopic follow-up, is complicated.

In conclusion, metachronous GISTs and adenocarcinoma in the colon are rare. However, it is important to carefully investigate and differentiate between potential lesions during a routine postoperative colonoscopy following colorectal cancer surgery, as the patient may present with rare GISTs, which may be confused with the recurrence of colorectal cancer. In addition, a surgical resection may be necessary, when the diagnosis of new neoplastic lesions during the follow-up colonoscopy of a colonic cancer patient is in doubt.
